# Neocortical layer 5 subclasses: From cellular properties to roles in behavior

**DOI:** 10.3389/fnsyn.2022.1006773

**Published:** 2022-10-28

**Authors:** Sara Moberg, Naoya Takahashi

**Affiliations:** ^1^Einstein Center for Neurosciences Berlin, Berlin, Germany; ^2^University of Bordeaux, CNRS, Interdisciplinary Institute for Neuroscience, IINS, UMR 5297, Bordeaux, France

**Keywords:** neocortical layer 5, pyramidal neuron, projection type, sensory processing, rodent behavior

## Abstract

Layer 5 (L5) serves as the main output layer of cortical structures, where long-range projecting pyramidal neurons broadcast the columnar output to other cortical and extracortical regions of the brain. L5 pyramidal neurons are grouped into two subclasses based on their projection targets; while intratelencephalic (IT) neurons project to cortical areas and the striatum, extratelencephalic (ET) neurons project to subcortical areas such as the thalamus, midbrain, and brainstem. Each L5 subclass possesses distinct morphological and electrophysiological properties and is incorporated into a unique synaptic network. Thanks to recent advances in genetic tools and methodologies, it has now become possible to distinguish between the two subclasses in the living brain. There is increasing evidence indicating that each subclass plays a unique role in sensory processing, decision-making, and learning. This review first summarizes the anatomical and physiological properties as well as the neuromodulation of IT and ET neurons in the rodent neocortex, and then reviews recent literature on their roles in sensory processing and rodent behavior. Our ultimate goal is to provide a comprehensive understanding of the role of each subclass in cortical function by examining their operational regimes based on their cellular properties.

## Introduction

The mammalian neocortex is a collection of functionally distinct circuits that are heavily interconnected and operate in parallel. Although each region of the neocortex serves different tasks, they share basic organizational rules, including a six-layer columnar structure and a diversity of cell types that comprise each layer. The main output of cortical columns is localized in layer 5 (L5), which consists of intermingled, non-overlapping populations of pyramidal cells, namely intratelencephalic (IT) and extratelencephalic (ET) neurons. L5 IT neurons project to other cortical areas and the striatum bilaterally. In contrast, ET neurons project to subcortical areas, including the ipsilateral striatum, higher-order thalamic nuclei, superior colliculus (SC), and pons. These projection patterns of IT and ET neurons can be further segregated into additional subcategories (Kim et al., 2015; Rojas-Piloni et al., 2017; Economo et al., 2018; Tasic et al., 2018). Here, we focus on IT and ET neurons as a whole, outlining the differences in their cellular properties and roles in sensory-motor processing and behavior. The following work mentioned in this review is mostly derived from rodents (mice and rats), unless stated otherwise.

## Anatomical and morphological properties

**FIGURE 1:**
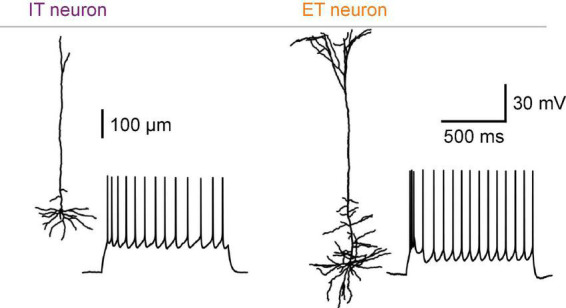
Intratelencephalic (IT) and extratelencephalic (ET) neurons in neocortical L5. Morphological and electrophysiological differences between IT and ET neurons in the mouse S1. Adapted from Takahashi et al. (2020).

IT neurons have relatively small cell bodies in the upper half of L5 (L5a), whereas ET neurons with large cell bodies are found primarily in lower L5 (L5b) (Wise and Jones, 1977; Kasper et al., 1994). One of the most prominent morphological features of L5 pyramidal neurons is the vertically extended dendritic tree, the so-called apical dendrite, which arises from the cell body and reaches the cortical surface (Figure 1). ET neurons have a thick apical dendrite (large diameter) with many distal branches extensively arborizing within layer 1 (L1) (Hattox and Nelson, 2007; Groh et al., 2010). Additionally, ET neurons have oblique branches protruding from the proximal trunk of the apical dendrite (Kasper et al., 1994; Groh et al., 2010). In contrast, IT neurons have a thin apical dendrite (small diameter) with poorly branched distal branches and few oblique dendrites (Kasper et al., 1994; Hattox and Nelson, 2007; Groh et al., 2010).

## Synaptic connectivity

As the neocortex consists of six layers, each of which receives different inputs from local circuits or distant brain areas, the anatomical and morphological difference between IT and ET neurons already implies differences in inputs that they primarily process. For example, L4 and L5b in the primary somatosensory cortex (S1) receive feed-forward sensory signals from the ventral posteromedial (VPM) thalamus—the primary thalamic nucleus that relays somatosensory information from the peripheries to the cortex. VPM thalamic axons terminate on the basal dendrites of ET neurons, feeding ET neurons with early somatosensory information (Constantinople and Bruno, 2013). In contrast, long-range feedback (top-down) projections from other cortical areas and higher-order thalamic nuclei are abundant in L1, where ET neurons have extensively-arborized distal branches while IT neurons have less. Notably, some feedback projections also target L5a, where they terminate at the somata and basal dendrites of IT and ET neurons (Petreanu et al., 2009; Groh et al., 2010; Mao et al., 2011). Kim et al. (2015) used monosynaptic rabies tracing to compare the long-range inputs to IT and ET neurons. They found that ET neurons receive more input from the frontal cortical regions that are implicated in top-down modulation of brain states (Zhang et al., 2014). Notably, cortico-cortical feedback inputs preferentially target IT neurons projecting back to the source area over IT neurons projecting to other cortical regions (Young et al., 2021). IT neurons thereby form looped circuits which facilitate recurrent interactions. Differences in embedded synaptic circuitry between IT and ET neurons are also evident locally within cortical columns. For each subclass, neurons are locally interconnected with other neurons in the same subclass. Interestingly, IT neurons connect to ET neurons, but ET neurons do not connect back to IT neurons (Brown and Hestrin, 2009; Kiritani et al., 2012), indicating unidirectional information flow between the two neuron types.

## Electrophysiological properties

Electrophysiological properties of IT and ET neurons have been extensively characterized by brain slice experiments (Figure 1). As discussed in the previous section, IT and ET neurons can be distinguished by their distinct morphological and anatomical features. Here we included studies where authors separate neuron types based on depth and morphology, thin-tufted L5a and thick-tufted L5b neurons, which are referred to as IT and ET neurons in this section, respectively.

Regarding somatic excitability and action potentials (APs), IT neurons have higher input resistance, higher firing thresholds, and higher rheobase than ET neurons (Mason and Larkman, 1990; Kasper et al., 1994; Hattox and Nelson, 2007). IT neurons are classified as regular spiking neurons and exhibit pronounced adaptation of the AP frequency in response to a depolarizing current pulse (Chagnac-Amitai et al., 1990; Mason and Larkman, 1990; Kasper et al., 1994; Hattox and Nelson, 2007) (Figure 1, left). A large slow afterhyperpolarization (sAHP) conductance is responsible for the AP adaptation in IT neurons (Guan et al., 2015). Individual APs of IT neurons are relatively broad in time due to a slow rate of the repolarization process (Mason and Larkman, 1990).

In contrast, ET neurons are distinguished by the tendency for their APs to appear in high-frequency bursts—intrinsically bursting (Chagnac-Amitai et al., 1990; Mason and Larkman, 1990; Kasper et al., 1994; Hattox and Nelson, 2007). In response to a current pulse, ET neurons respond with a brief burst (often seen as an AP doublet) followed by repetitive single non-adapting APs (Figure 1, right). ET neurons have very little sAHPs, which may explain their non-adapting firing pattern (Mason and Larkman, 1990; Guan et al., 2015). Individual APs of ET neurons are narrower and accompanied by more significant fast and medium AHPs (fAHPs and mAHPs, respectively) and afterdepolarizations (ADPs) than those of IT neurons (Hattox and Nelson, 2007). The difference in polarization and repolarization mechanisms between the two subclasses may also impact the firing modes, explaining why ET neurons are more prone to burst firing.

Another notable difference between the two subclasses is a hyperpolarization-activated cation current (*I*_h_), measured as a “sag” potential in response to hyperpolarization. ET neurons show a pronounced sag response to hyperpolarizing currents, whereas IT neurons show little sag response (Mason and Larkman, 1990; Kasper et al., 1994; Hattox and Nelson, 2007). *I*_h_ currents are mediated by hyperpolarization-activated cyclic nucleotide-gated non-selective cation (HCN) channels and prevent the membrane potential from being too hyperpolarized. HCN channels are tonically active near the resting membrane potential, and thus act as a leak conductance that impedes the accumulation of synaptic potentials (Biel et al., 2009). Interestingly, HCN channels are expressed in a gradient along the somatodendritic axis of ET neurons and are more abundant in distal dendrites (Berger et al., 2001; Lorincz et al., 2002; Kole et al., 2006; Harnett et al., 2015). The impact of *I*_*h*_ conductance on dendritic excitability and synaptic integration is discussed in the next section.

## Synaptic integration in dendrites

Both L5 IT and ET neurons have an apical dendrite, which electrically bridges between the distal dendrites and somata and plays an essential role in regulating neuronal excitability. Here we discuss the differences in the integration of synaptic inputs at the apical dendrites between IT and ET neurons. The classical view on synaptic integration in a neuron is that synaptic potentials from input sites passively propagate along dendritic arbors to the AP initiation site—axon initial segment (Rall, 1959, 1967). The amplitude of a synaptic potential declines exponentially with travel distance and branching points. The long, vertically elongated apical dendrites of L5 pyramidal neurons therefore function as an electrical filter that attenuates distal inputs and prevents them from affecting somatic APs. By simultaneous whole-cell recording of the soma and apical dendrite of single L5 neurons in slices, Williams and Stuart demonstrated that synaptic potentials at the distal dendrites are attenuated approximately 40-fold by the time they reach the soma (Williams and Stuart, 2002). The attenuation is even more severe in more realistic *in vivo* conditions, where dendrites are bombarded with spontaneous synaptic inputs that shunt out currents (Bernander et al., 1991; Williams, 2004).

Additionally, *I*_*h*_ conductances spur attenuation of synaptic potentials in dendrites. In ET neurons, due to the dendritic gradient of the HCN channel density, *I*_*h*_ conductances severely impact the summation and propagation of synaptic potentials in the distal apical dendrites (Berger et al., 2001; Harnett et al., 2015). The differences in morphology and HCN channel expression between IT and ET dendrites imply their distinct filtering properties for processing distal inputs. In the medial prefrontal cortex (mPFC), Dembrow et al. (2015) have indeed shown that the apical dendrites of IT and ET neurons exhibit distinct subthreshold voltage responses to identical synaptic inputs. They found that the amplitude of synaptic potentials attenuated similarly between the subtypes, but the time courses of the transferred potentials were significantly stretched in IT neurons. The difference in the time window for input summation suggests that ET dendrites act more as coincidence detectors while IT dendrites act as temporal integrators. Nevertheless, transferred synaptic potentials from distal dendrites are still very small at the soma, failing to elicit APs. Small voltage fluctuations in distal dendrites are therefore electrically segregated from the soma in both IT and ET neurons.

## Active dendritic properties

The distal dendrites in L1 are innervated extensively by feedback projections from various cortical areas and higher-order thalamic regions. How can these inputs influence the somatic output, given the strong attenuation they undergo? Dendrites not only passively integrate or transmit electrical signals, but can also actively process incoming inputs and transform them into AP outputs. The regenerative activation of voltage-gated ion channels, which are distributed throughout the dendritic membrane, can lead to the generation of spikes in dendrites, so-called dendritic spikes. For example, somatically-generated APs can propagate back into the apical dendrite with little attenuation through activation of dendritic sodium channels along the apical trunk, so-called back-propagating APs (bAPs) (Stuart and Sakmann, 1994; Stuart et al., 1997). bAPs provide a rapid retrograde signal that could modulate membrane excitability of the distal dendrites and interact with synaptic inputs to regulate dendritic plasticity. Although bAPs are seen in both IT and ET neurons, their propagation appears less efficient in IT neurons, probably due to a lower density of active conductances in the apical dendrite (Grewe et al., 2010). In addition to voltage-sensitive sodium channels, apical dendrites also express various types of voltage-sensitive calcium channels, including both low- and high-threshold channels (Perez-Garci et al., 2013). The regenerative activation of these channels causes long-lasting plateau potentials, so-called dendritic calcium spikes (Amitai et al., 1993; Yuste et al., 1994; Schiller et al., 1997; Larkum et al., 1999b). Calcium spikes are generated at the apical trunk regions and propagate forward to the soma with little attenuation and strongly depolarize the somatic membrane, leading to bursts of high-frequency APs (Larkum et al., 1999b; Williams and Stuart, 1999). Synchronized inputs to distal dendrites or a train of high-frequency bAPs can trigger calcium spikes (Schiller et al., 1997; Larkum et al., 1999a). Larkum et al. (1999b) have demonstrated that distal inputs coincided with bAPs efficiently elicit calcium spikes, which are therefore suggested as coincidence detectors for correlating feedback inputs arriving at the distal dendrites with feed-forward sensory inputs at the soma. Responding to varying distal inputs, calcium spikes also serve as a mechanism for modulating the gain of the somatic AP output by changing the firing mode from isolated spikes to bursting (Larkum et al., 2004). Thus, calcium spikes are essential for integrating distal input with somatic activity. It should be noted that most studies on calcium spikes have been conducted on ET neurons. We still know very little about calcium spikes in IT neurons, but recent studies indicate that they might have less active calcium conductances or have a higher threshold for the generation of calcium spikes compared to those in ET neurons (Grewe et al., 2010; Takahashi et al., 2020). Dembrow et al. (2015) measured the dendritic and somatic voltage responses of IT and ET neurons in mPFC to depolarizing currents injected into the apical dendrites. Interestingly, while isolated distal synaptic inputs fail to propagate to the soma, the strong depolarizing currents reliably elicited calcium spikes in the apical dendrites of ET neurons, which drove burst firing at the soma. In contrast, in IT neurons, the injected currents failed to generate calcium spikes but directly triggered somatic APs.

## Neuromodulation

The differences between IT and ET neurons include their response to neuromodulatory inputs. Neuromodulatory projections typically extend throughout the neocortex, but most work on the influence of neuromodulators has concentrated on the mPFC circuit due to its involvement in higher-order cognitive functions. We therefore present work mainly from mPFC, if not stated otherwise, but it is important to keep in mind that neuromodulatory effects may differ between brain regions. Here, we focus on dopamine (DA), acetylcholine (ACh), norepinephrine (NE), and serotonin (5-HT) as these neuromodulators have shown to exert distinct effects on IT and ET neurons due to differential expression of each respective receptor subunit and subtypes.

### Dopamine

Dopaminergic projections from the ventral tegmental area predominantly target cortical pyramidal neurons in L5 due to their innervation patterns in the lower layers of the cortex (Descarries et al., 1987; Nomura et al., 2014). DA receptors can be segregated into two kinds of G-protein-coupled receptors: D1-receptor and D2-receptor. Differential expressions of DA receptors are found between IT and ET neurons in PFC (Seong and Carter, 2012). D1-receptors were found to be predominantly expressed in L5 neurons with little *I*_*h*_ current, putative IT neurons, but activation of these receptors only slightly enhanced their excitability (Xing et al., 2021). D2-receptors, however, were found predominantly on ET neurons (Gee et al., 2012) and activation of D2-receptors on these neurons drives calcium channel-dependent ADPs, leading to sustained repetitive firing. How this selective enhancement of excitability in IT and ET neurons upon DA release in the cortex relates to separate functional pathways remains unknown.

### Acetylcholine

In contrast to dopaminergic inputs, cholinergic fibers from the basal forebrain innervate the cortex in both L1 and L6 (Bloem et al., 2014) and there’s a plethora of evidence suggesting differential responses in IT and ET neurons and a central role of ACh in cognitive functions such as arousal and attention (Froemke et al., 2007; Nunez et al., 2012; Letzkus et al., 2015; Herrero et al., 2017; Baker et al., 2018). Overall, IT neurons exhibit only a slight enhancement in firing in response to ACh compared to ET neurons that shows a robust persistence in firing (Baker et al., 2018). ACh acts through two different kinds of receptors, the slower G-protein-coupled muscarinic receptors (mAChR) and the faster ionotropic nicotinic receptors (nAChR). mAChRs express on both IT and ET neurons and exert somewhat similar effects on each type; an initial transient hyperpolarization followed by a prolonged depolarization (Baker et al., 2018). However, the mAChR-mediated inhibition is more pronounced in IT neurons, while excitatory responses are greater and longer lasting in ET neurons, which can lead to persistent firing (Joshi et al., 2016). Recent work has shown that local ACh release enhances calcium spikes in the apical dendrites of ET neurons *via* activation of R-type calcium channels mediated by mAChRs (Williams and Fletcher, 2019), converting ET neurons from regular to burst firing mode (Nunez et al., 2012). Moreover, Suzuki and Larkum (2020) showed that mAChRs are necessary for dendro-somatic coupling in L5 pyramidal neurons in S1. Overall, transient ACh release preferentially promotes ET neuronal firing over IT neurons *via* mAChR activation.

Nicotinic receptors consist of a combination of α and β subunits. The predominant nAChRs found in sensory cortices are hetero-pentameric receptors composed of α4 and β2 subunits and homo-pentameric receptors composed of α7 subunits (Gotti et al., 2009). In the rodent neocortex, local ACh application or activation of cholinergic fibers evokes nAChR-mediated, transient excitatory currents in L5 neurons (Zolles et al., 2009), in both IT and ET neurons (Joshi et al., 2016). Interestingly, L5b (putative ET) neurons in the rat primary auditory cortex (A1) exhibit an age-related decrease in ACh sensitivity due to a shift in the nAChR subunit composition with an increase in the α7 subunit (Ghimire et al., 2020), which has lower ACh affinity (Fenster et al., 1997).

### Norepinephrine

NE is released from the locus coeruleus (LC), which projects to many areas, including the neocortex (Loughlin et al., 1986; Nomura et al., 2014), and has an essential role in sensory processing and arousal (Bouret and Sara, 2002; Sara, 2009; Carter et al., 2010; Janitzky et al., 2015; Hayat et al., 2020). Noradrenergic fibers generally spread across all layers of the cortex (Nomura et al., 2014), providing a broader blanket of modulation. Somewhat similar effects of NE have been observed in IT and ET neurons, where IT neurons showed a slight increase in AP firing and ET neurons exhibit a greater increase (Dembrow et al., 2010). Activation of α2A adrenergic receptors strengthens working memory through inactivating cAMP, leading to the closure of HCN channels in rodents and monkeys (Franowicz et al., 2002; Wang et al., 2007). Moreover, this was found to dominantly affect the apical dendrites of L5 pyramidal neurons in PFC, where α2A adrenergic receptor activation reduces the threshold for which bAPs can trigger dendritic calcium spikes leading to increased dendritic excitability (Barth et al., 2008; Labarrera et al., 2018). Using two-photon calcium imaging *in vivo*, Labarrera et al. (2018) showed that activation of α2A adrenergic receptors increases the dendro-somatic coupling through blockage of *I*_h_.

### Serotonin

Serotonergic afferents arrive in the neocortex mainly from the dorsal raphe nucleus (Bang et al., 2012). The effect exerted on different neuron types differs depending on the composition of receptor subtypes on the postsynaptic neuron. 5-HT receptors are classified into seven main groups, but L5 pyramidal neurons mainly express a combination of 5-HT_1A_ and 5-HT_2A_ receptors (Amargós-Bosch et al., 2004). Generally, activation of 5-HT_1A_ receptors has an inhibitory effect, while 5-HT_2A_ receptor activation has an excitatory effect. 5-HT receptors on ET neurons in mice consist of predominantly inhibitory 5-HT_1A_ receptors, and application of 5-HT to mPFC ET neurons leads to an increase of *I*_h_ currents (Avesar and Gulledge, 2012; Stephens et al., 2014; Elliott et al., 2018). 5-HT could therefore have an opposite effect on the excitability of ET neurons compared to ACh or NE, indicating a potential competitive mechanism to regulate attention and arousal in different contexts. In contrast, IT neurons consist of a combination of 5-HT_1A_ and 5-HT_2A_ receptors and 5-HT application typically causes depolarization (Avesar and Gulledge, 2012; Stephens et al., 2014; Elliott et al., 2018). This can either be direct or biphasic following a brief inhibitory 5-HT_1A_ activation followed by the excitatory 5-HT_2A_ effect.

Overall, the neuromodulatory effects of 5-HT are strikingly different in IT and ET neurons regardless of brain regions, although inconsistencies exist between cell types and species (Elliott et al., 2018). Further work is needed to elucidate how these differences relate to the IT-ET interplay during different contexts and conditions.

## Computational roles

**FIGURE 2:**
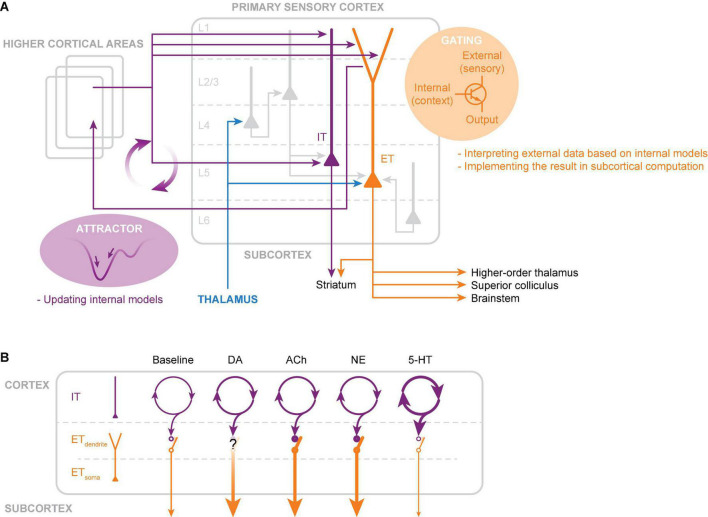
Hypothesized circuits and computational roles of intratelencephalic (IT) and extratelencephalic (ET) neurons in L5. **(A)** IT neurons constitute a dense recurrent network within and across cortical areas. For sensory processing, the IT network integrates external sensory evidence and seeks stable states (attractors) in activity space through synaptic connections formed by past experience. Each attractor represents an internal model, with which the brain compares against incoming sensory data. Internal models are constantly updated and fed into the distal apical dendrites of ET neurons. These feedback inputs serve as gating signals that selectively enhance the output of ET neurons for sensory inputs that match with the internal model. Boosted ET outputs are broadcasted to the subcortical structures, which integrate this filtered sensory information. **(B)** Simplified schematic illustration of various neuromodulation acting differently on IT somatic and ET dendritic and somatic activities. Note that the illustration demonstrates the net effect of individual neuromodulators (see the Section on “Neuromodulation” for effects on various receptor subtypes and temporal dynamics). The thickness of the lines represents changes in activity due to neuromodulation (thicker lines indicate increased activity and thinner lines indicate decreased activity).

We have so far discussed the morphological, anatomical, and physiological properties of IT and ET neurons in neocortical L5, as well as the neuromodulatory influence on these subtypes. It is evident that in each one of these aspects, there are clear differences between IT and ET neurons. The computational roles of IT versus ET neurons have been discussed by Harris and Shepherd (2015). They suggest that ET neurons act as downstream elements in the local circuit, which integrates the results of local computations with direct thalamic inputs and efficiently broadcasts the results to distant subcortical structures. In contrast, local and long-range connectivity of IT neurons forms the backbone of communication within and between cortical areas and hemispheres. Their outputs go to other cortical areas and striatum, as well as locally to ET neurons. Here, we build on these insights and provide our perspective on the computational role of IT and ET neurons in sensory processing by taking into account the differences in their physiological and anatomical properties (Figure 2).

IT neurons form a dense recurrent network through their local and long-range connections. In such network configurations, activity patterns converge to stable states (fixed points) in activity space (repertoire of possible activity patterns), known as attractor dynamics. Attractor networks have been proposed as a model of cortical learning and memory (Hopfield, 1982; Amit and Brunel, 1997). The synaptic connectivity between neurons determines the activity space and its multiple attractor points. Each attractor state thus represents a stored memory, which reflects an internal model held within the cortex—inner belief of how the world should be or representation of behavior context and task rules (Yang et al., 2019). Based on available sensory data, attractor networks construct probabilistically plausible internal models. The IT neuronal network is well-suited for building such internal models based on experience and learning. Interestingly, a computational study indicates that the spike frequency adaptation, an electrophysiological characteristic of IT neurons, can allow the network to integrate temporally dispersed information seamlessly into ongoing network activity (Salaj et al., 2021). In sensory information processing, internal models are essential for interpreting incoming sensory input, such as detecting deviations from expectations, filling in missing information, and assigning weights to information relevant to tasks and contexts (Gilbert and Sigman, 2007). Neuromodulators such as 5-HT regulate the intrinsic excitability and synaptic integration of IT neurons. Such neuromodulatory influence can enhance the capability and flexibility of computation in the IT neuronal network, dynamically adapting the internal model to diverse behavioral demands (Tsuda et al., 2022). Lastly, but perhaps most importantly, internal models generated by the IT neurons are fed back to ET neurons.

ET neurons receive broadly-tuned sensory information mainly at the basal dendrites, while non-sensory (feedback) information, mostly from IT neurons in distant cortical areas, targets the distal apical dendrites. Due to the electrical distance between the distal dendrites and the soma, feedback inputs alone have little influence on the somatic AP output. Feedback inputs act as a filter that gates coincidental sensory inputs arriving at the soma through activating calcium spikes in the apical dendrites. The spatial and temporal characteristics of the filter depend on the target specificity of the feedback inputs and their duration, respectively. The spatiotemporal characteristics are further shaped by the intrinsic properties of the dendritic membrane. Finally, gated sensory information is boosted into bursts of APs and transmitted to the subcortical regions. It is intriguing that neuromodulators such as ACh and NE, which are elevated in arousal and alert states, promote calcium spikes in ET neurons, thereby enhancing the interaction between feedback and sensory information. As a result, the internal model could edit or highlight specific cortical representations of the sensory environment and output the results to subcortical structures to exert control over subcortical sensory-motor processing.

Interestingly, due to the lack of connection from ET to IT neurons, the gated ET output does not directly influence the activity of local IT neurons. Thus, internal models are retained in IT neurons without being affected at a local scale. Although there is no direct interference from ET to IT neuronal activity, the ET output to subcortical regions could influence the activity of IT neurons *via* subcortical-to-cortical ascending pathways. One such pathway is thalamocortical projections *via* the higher-order thalamus (Sherman, 2016), which have diffusive innervation in the cortex. Thus, in principle, the output of gated ET neurons can interfere with the activity of IT neurons, not locally but rather on a global scale, which may promote a transition in attractor states in IT neuronal activity.

In summary, these two subclasses of L5 pyramidal neurons are likely to play distinct roles in cortical computation, leading to the natural hypothesis that they are involved in different brain functions. Next, we discuss what role each subclass of L5 pyramidal neurons plays in sensory processing and behavior.

## Sensory processing

The following body of work mainly separates ET and IT neurons depending on the depth of the somata. Here, we generally consider the neurons in L5b to be the ET neurons and neurons in L5a to be IT neurons.

ET neurons in L5b have slightly more spontaneous activity than IT neurons in L5a (Manns et al., 2004). Differences in sensory receptive fields of IT and ET neurons have been well-studied. For instance, IT neurons seem to have a narrower receptive field to passive stimulus and a rapid onset of response in contrast to ET neuronal responses which have broader receptive fields and slow onset of response (Palmer and Rosenquist, 1974; Finlay et al., 1976; Manns et al., 2004). During whisking without object contact, IT neurons in S1 significantly increase their firing compared to ET neurons (de Kock and Sakmann, 2009). In contrast, during active whisker touch, a subset of ET neurons (but not IT neurons) goes into burst firing, which could reliably infer the touch event (de Kock et al., 2021). Burst firing of ET neurons could therefore convey relevant touch information of the surrounding environment to subcortical targets. The reasons for these separate sensory response properties and how they relate to animal behavior will be discussed in the next section.

## Behavior

In recent years, there has been a surge of evidence of the distinct functional roles of IT and ET neurons in cognitive behavior (Li et al., 2015; Takahashi et al., 2020; Bae et al., 2021; Currie et al., 2021; Musall et al., 2021; Heindorf and Keller, 2022; Mohan et al., 2022; Park et al., 2022). With recent development of combining whole-brain imaging with newly developed mouse lines, it is now possible to record brain-wide activity of IT neurons (including both L5 and L2/3 IT neurons) and L5 ET neurons. It seems that IT neurons are strongly engaged in preparatory stages of various tasks as they are more active in sensory regions during sensory sampling phases (Musall et al., 2021; Mohan et al., 2022). This trend is consistent in other areas such as mPFC where they retain goal specific responses prior to action initiation (Bae et al., 2021) and preparatory movement activities in motor structures (Currie et al., 2021; Heindorf and Keller, 2022). Inhibiting the IT neurons during sampling periods only weakly affects the behavioral outcome, suggesting a minor role in regulating behavioral output.

ET neurons are instead engaged at later stages such as decision phases and during action initiation (Bae et al., 2021; Currie et al., 2021; Musall et al., 2021; Mohan et al., 2022). They have been found to be more prominent in motor structures where they drive movement initiation (Musall et al., 2021; Mohan et al., 2022). However, ET neuronal activity can also be seen in sensory areas where they regulate detection of behaviorally-relevant sensory stimuli (Ruediger and Scanziani, 2020; Takahashi et al., 2020). Thus, this reinforces the concept of IT neurons maintaining and updating cortical neurons for more efficient computations and ET neurons broadcasting this updated information to the subcortical structures. With the recent advancements of modern tools, we have come a long way to perturb IT and ET neuronal circuits. There is a barrage of work in recent years showing specific roles of distinct circuits of IT and ET neurons but efforts to delineate the multiple circuits of IT and ET neurons and compare those across each other remains extremely difficult. As the tools and technology advances, so does our ability to probe these circuits and our understanding on how they relate to each other. Although evidence of separate L5 IT neuronal circuits in behavior remains limited, more work has been done on ET neurons and their specific downstream targets, presenting rich segregation of roles during behavior. Next, we elaborate further on these distinct differences in target-selective ET neurons.

### Superior colliculus

SC-targeting ET neurons are more sensitive to contrast thresholds, thus modulating the sensitivity to detect relevant sensory stimuli (Lur et al., 2016; Ruediger and Scanziani, 2020; Takahashi et al., 2020). Using two-photon imaging, Takahashi et al. (2020) recorded the activity from the apical dendrites in L1 of ET neurons. They showed that dendritic calcium spikes dynamically regulate sensory responses in ET neurons in S1 for tactile detection in a context-dependent manner. Local chemogenetic inactivation of the ET neuronal output in SC shifted the mouse’s perceptual threshold toward higher intensities, indicating the importance of the corticocollicular pathway in detecting sensory stimuli relevant to a given context. SC is an excellent structure for integrating multiple sensory modalities (May, 2006; Zingg et al., 2017) from both cortical and peripheral sources *via* the brainstem (Huerta et al., 1983; Jacquin et al., 1989; Adibi, 2019). SC could therefore function as a multisensory integrator for guiding behavior by comparing the contextually important signals transmitted by ET neurons with the incoming peripheral sensory inputs.

### Pons

The pons receives extensive cortical input from ET neurons and projects selectively to the cerebellum, serving as an essential node for cortico-cerebellar communication. Perturbation of the ponto-cerebellar pathway does not affect movement initiation, but disrupts the success rate and precision of the movement (Guo et al., 2021). Using mice trained for a variable forelimb movement task with a joystick, Park et al. (2022) showed that pons-projecting ET neurons in the primary motor cortex (M1) represent movement direction. Interestingly, in the same behavior, striatum-projecting IT neuronal activity in M1 was preferentially tuned to movement amplitude, with little to movement direction. In the primary visual cortex (V1), pons-projecting ET neurons were found to be engaged in a visually-cued conditioned eyeblink task (Tang and Higley, 2020), indicating a role for the cortico-ponto-cerebellar pathway in reflexive motor responses (Freeman and Steinmetz, 2011). In contrast, a similar sensory detection task, but requiring goal-directed, non-directional movements, does not require the output of ET neurons in the sensory cortex to pons (Takahashi et al., 2020). These results highlight the role of pons-projecting ET neurons in specific aspects of motor control.

### Higher-order thalamus

Higher-order thalamic projections seem to be of an interesting class of ET neurons. Single-cell reconstructions of axons of these ET neurons reveal clusters of axon terminals with large boutons (Hoogland et al., 1987; Bourassa and Deschênes, 1995). Such giant corticothalamic terminals, so-called “driver synapses,” elicit large synaptic potentials and can drive spiking of postsynaptic neurons in the higher-order thalamus (Reichova and Sherman, 2004; Mease et al., 2016b). Repetitive activation of these synapses results in strong short-term depression, narrowing the temporal window for transmitting activity from the cortex to the thalamus (Li et al., 2003; Groh et al., 2008).

In the somatosensory system, ET neurons in S1 form driver synapses on neurons in the posterior medial nucleus (PoM) of the thalamus. A recent study demonstrated that PoM-targeting ET neurons regulate the mouse’s perceptual threshold for detecting tactile stimuli (Takahashi et al., 2020). Importantly, PoM sends its projections back to S1, where they preferentially target IT neurons (Petreanu et al., 2009), forming a recurrent loop between the cortex and thalamus (Wimmer et al., 2010; Mease et al., 2016a; Guo et al., 2020). This recurrent loop may also serve to strengthen the coupling between apical dendrites and soma of L5 neurons (Suzuki and Larkum, 2020).

The motor cortical areas also form a strong recurrent loop with higher-order motor thalamic nuclei. In a delayed motor task, the loop between the anterior lateral motor cortex (ALM) and the motor thalamus builds persistent preparatory activity reflecting upcoming behavioral choices (Guo et al., 2017). Thalamus-targeting ET neurons in ALM therefore play a crucial role in maintaining the premotor activity in the loop.

### Striatum

The striatum is a unique structure in the sense that it is innervated by both IT and ET neurons, albeit bilaterally versus unilaterally, respectively. Interestingly, in the striatum, axon terminals of IT neurons are characteristically small (0.4–0.5 μm) compared with those of ET neurons (0.8–0.9 μm) (Reiner et al., 2003). This may indicate a difference in the nature of their influence on the striatal activity and an intriguing contrast in their roles during behavior. In the motor system, striatal-targeting IT neurons have been shown to regulate amplitude and speed of goal-directed forelimb movements (Park et al., 2022). Moreover, the unidirectional IT-to-ET neuronal connectivity supports the role of IT neurons modulating ET neuronal output and preparing action initiation (Guo et al., 2021). In the sensory system, during an auditory discrimination task, striatal-targeting IT and ET neurons originating from A1 drive behavioral choices (Znamenskiy and Zador, 2013). The engagement of striatal-targeting IT and ET neurons is, however, absent during sensory detection or pronounced only to a small extent (Ruediger and Scanziani, 2020; Takahashi et al., 2020), indicating the importance of this pathway for feature discrimination but not for sensory detection. On the contrary to the abovementioned dichotomic roles, there is strong evidence of their participation during sensory associative learning (Xiong et al., 2015; Ruediger and Scanziani, 2020). Further investigations are necessary to delineate the role of IT-striatal versus ET-striatal pathway in cognitive processes.

## Conclusion

This review has highlighted differences in morphological, anatomical, and physiological properties between IT and ET neurons, as well as in neuromodulatory effects on cellular excitability. Based on these differences, we hypothesized a distinct role for IT and ET neurons in cortical sensory processing: IT neurons generate an internal model, which acts as a spatiotemporal filter that gates the output of a selected population of ET neurons to the subcortical structures. With recent methodological advances, such as genetic targeting, population recording and optogenetics, it has now become possible to investigate their modes of operation in the living brain and even during behavior. As a result, distinct modes of operation between subtypes in sensory and motor processing are becoming evident, each impacting behavior in different ways. However, it remains to be determined how the morphological and physiological characteristics contribute to the *in vivo* operation of each subtype during behavior (Takahashi et al., 2020; Otor et al., 2022). The highlighted difference between IT and ET neurons, as well as the hypothesized computational roles for each subtype, in this review may be useful to facilitate both experimental and theoretical studies to gain a mechanistic understanding of L5 subcircuits in cortical information processing.

In this study, we reviewed IT and ET neurons in neocortical L5, focusing on rodent studies. Recently, attempts have begun to characterize human L5 neurons physiologically, morphologically and genetically using surgically resected human cortical tissue. These studies have revealed many conserved properties of rodent and human L5 neurons, supporting the translational relevance of the findings in rodents, while some divergent properties have also been identified (Beaulieu-Laroche et al., 2018, 2021; Kalmbach et al., 2021). For example, compared to rodents, distal inputs provide very little excitation to the soma in human ET neurons even in the presence of dendritic spikes (Beaulieu-Laroche et al., 2021). These examples emphasize the need for continued research identifying conserved and specialized properties of L5 neuronal subtypes in humans and possibly in other animal species. The future studies would provide deeper insights into the fundamental and universal role of L5 neurons in cortical computation, and would also highlight phenotypic divergence related to cortical functions specialized to individual animal species.

## Author contributions

Both authors wrote the manuscript and approved the submitted version.
